# Hsa_circ_0002238 promotes the malignant behavior of colorectal cancer

**DOI:** 10.3389/fphar.2025.1541820

**Published:** 2025-06-13

**Authors:** Lin-Ting Zhang, Yuejia Zhou, Tiantian Wang, Boran Chen, Zhigao Cai, Shubin Wang, Gangling Tong

**Affiliations:** ^1^ Department of Oncology, PKU-Shenzhen Clinical Institute of Shantou University Medical College, Shenzhen Key Laboratory of Gastrointestinal Cancer Translational Research, Cancer Institute of Shenzhen-Peking University-Hong Kong University of Science and Technology (PKU-HKUST) Medical Center, Shenzhen, Guangdong, China; ^2^ Department of Gastrointestinal Surgery, Longchuan County People’s Hospital, Heyuan, Guangdong, China

**Keywords:** circular RNA, colorectal cancer, proliferation, migration, invasion, epithelial-mesenchymal transition, apoptosis

## Abstract

**Background:**

Colorectal cancer (CRC) is one of the most common and deadly cancers worldwide. Circular RNAs (circRNAs) have emerged as crucial players in the onset and progression of CRC. This research aims to investigate the expression levels of a novel circRNA, hsa_circ_0002238, in CRC and to explore its association with alterations in CRC functional phenotypes.

**Methods:**

High-throughput RNA sequencing identified abnormal circRNA expressions, and qRT-PCR validated hsa_circ_0002238 expression. The relationship between hsa_circ_0002238 expression and the clinical data was analyzed by student’s t-test. Fluorescence *in situ* hybridization determined its cellular localization and expression in CRC cells. was conducted to ascertain the specific localization of hsa_circ_0002238 within cells and further confirm its expression in CRC cells. Following transfection with siRNA or plasmid, CRC cell proliferation was evaluated by CCK-8 assays and apoptosis was assessed by flow cytometry. We assessed changes in proliferation capacity among CRC cells exhibiting high levels of hsa_circ_0002238 using CCK-8 assays and flow cytometry analysis. Furthermore, flow cytometry was also used to evaluate apoptosis rates in these high-expressing CRC cells. In addition, wound healing and transwell assays were performed to assess changes in migratory and invasive capabilities associated with elevated hsa_circ_0002238 expression. The study additionally conducted *in vivo* experiments to validate the impact of hsa_circ_0002238 on the growth of CRC cells. Finally, Western blot was employed to analyze the expressions of epithelial-mesenchymal transition (EMT), serine/threonine kinase (AKT)/phosphatidylinositol 3 kinase (PI3K) signaling pathway, and apoptosis-related molecules.

**Results:**

Our findings showed that hsa_circ_0002238 was significantly overexpressed in both CRC cell lines and tumor tissues. The expression level of hsa_circ_0002238 correlates with patient gender (p = 0.017) and shows significant diagnostic value (AUC = 0.765, 95%CI: 0.618–0.913, p = 0.004). At a relative expression level of 5.836, it achieves high sensitivity (50%) and specificity (100%). This upregulation promotes cellular proliferation, migration, invasion while inhibiting apoptosis within CRC cells. *In vivo* stuides, knockdown of hsa_circ_0002238 inhibited CRC tumor growth. Specifically, hsa_circ_0002238 facilitates EMT process characterized by markedly reduced E-cadherin levels alongside increased N-cadherin, vimentin and β-catenin expressions. Moreover, it induces elevated p-AKT and p-PI3K levels, increases cleaved caspase 3 and bcl-2, and decreases Bax expression in CRC cells, indicating that hsa_circ_0002238 enhances PI3K/AKT signaling and suppresses apoptosis. elevated p-AKT, levels along with cleaved caspase 3, bcl-2 expression within CRC, suggesting that hsa_circ_0002238 enhances AKT signaling and suppresses apoptosis.

**Conclusion:**

Our research demonstrates that hsa_circ_0002238 expression significantly enhances CRC proliferation, migration, invasion, EMT process, PI3K/AKT signaling pathway while inhibiting apoptosis. Additionally, a preliminary association between hsa_circ_0002238 levels and patient gender was found, suggesting its potential as a diagnostic marker for CRC.

## 1 Introduction

At present, colorectal cancer (CRC) ranks third in global incidence, accounting for approximately 9.6% of all cancer cases worldwide. Furthermore, the mortality rate associated with CRC is notably high, contributing to about 9.3% of total cancer deaths (Bray et al., 2024). Notably, the 5-year overall survival rate for patients diagnosed with early-stage CRC is around 91%, whereas it drops dramatically to about 14% for those with metastatic disease. This disparity underscores the poor prognosis faced by patients suffering from advanced stages of CRC (Siegel et al., 2023; Inoue et al., 2024). Therefore, early diagnosis of CRC is crucial. In recent years, non-coding RNAs have emerged as significant contributors to the early detection of CRC. For instance, 13 microRNAs (miRNAs) have been identified as being associated with KRAS mutations in CRC, indicating that these miRNAs may serve as potential biomarkers for early diagnosis (Wu et al., 2022). Furthermore, long non-coding RNAs (lncRNAs) ASMTL-AS1 and LINC02604 can facilitate CRC tumorigenesis by targeting mRNA through miRNA interactions, making them excellent diagnostic markers for CRC (Shakeri et al., 2024). Nevertheless, both miRNA and lncRNA are linear RNAs, which are easily degraded by RNase, and their clinical application are still limited.

Circular RNA (circRNA) is a novel class of non-coding RNA with longer half-life, which is formed through the back-fusion of linear RNAs or the back-splicing of pre-mRNA transcripts (Memczak et al., 2013). Compared to other types of RNA, circRNA possesses a closed-loop structure that confers remarkable stability against degradation by RNA hydrolysis (Feng et al., 2023). Studies have demonstrated that the functions of circRNAs are remarkably diverse. For instance, circRNAs can act as competitive sponges for miRNAs, thereby modulating gene expression (Zhang J. et al., 2024). They may also function as protein bait or facilitate protein translation (Zhang J. et al., 2024). Furthermore, circRNAs can undergo modifications through N6-methyladenosine, which plays a pivotal regulatory role in various biological processes associated with tumors (Wu et al., 2024; Zhang J. et al., 2024). CircRNAs exhibit aberrant expression patterns in CRC tissues, cells, exosomes, and blood-characteristics that underscore their high conservation and stability (Zhang J. et al., 2024). They mediate critical aspects of CRC pathogenesis, including tumorigenesis, metastasis, and drug resistance (Zhang J. et al., 2024). Consequently, circRNAs hold promise as both diagnostic and prognostic biomarkers for CRC and represent potential therapeutic targets in clinical settings. Research showed that hsa_circRNA_0000467 had been shown to facilitate the progression of CRC through enhancing c-Myc translation mediated by eIF4A3 (Jiang et al., 2024). In addition, the circ_0000395/miR-153-5p/MYO6 axis has been shown to promote cell growth, metastasis, and oxaliplatin resistance in CRC (Xiao et al., 2024). However, current research on circRNA related to CRC requires further investigation. This exploration could uncover additional novel circRNAs that may serve as molecular markers for the early diagnosis and prognosis of CRC, thereby providing new targets for future research and the development of innovative CRC therapeutics.

In previous research and analysis, we found that hsa_circ_0002238 was highly expressed in CRC tissues compared with the adjacent tissues through high-throughput sequencing. However, the specific function and diagnositic value of hsa_circ_0002238 in CRC is still unclear. Therefore, this study mainly discusses the specific function and diagnositic value of hsa_circ_0002238 in CRC, and provides a theoretical basis for the follow-up research.

## 2 Materials and methods

### 2.1 Cell culture and tissue sample collection

Human colorectal cancer HT-29, HCT116, SW480, LoVo cell line and human normal colon NCM460 cell line were purchased from Pricella company (Wuhan, China). HT-29 cells and NCM460 cells were cultured in RPMI-1640 medium (Gibco, Grand Island, NY, United States). LoVo cells were cultured in DMEM medium (Gibco, Grand Island, NY, United States). HCT116 cells were cultured in McCoy’s 5A medium (Pricella, Wuhan, Guangdong, China), while SW480 cells were cultured in Leibovitz’s L-15 medium (Pricella, Wuhan, Guangdong, China). All the cell lines were cultivated in the medium with 10% FBS (ExCell Bio, Shanghai, China) and in the environment under 5% CO_2_ (except SW480 cells), at 37°C and 80% humidity. A total of 20 paired CRC tissue samples were collected from Peking University Shenzhen Hospital between April and May 2024. All patients were over 18 years old and have been diagnosed with CRC based on pathological biopsy results. Written informed consent was obtained from all participants. Tumor staging was determined using the criteria established by the American Joint Committee on Cancer (8th edition) (Amin et al., 2017). Patients’ clinical characteristics data were extracted from their medical records and conducted in accordance with the principles of the Helsinki Declaration. All patients have signed the written informed consent form. This study received approval from the Ethics Committee of Longchuan County People’s Hospital [(2022) No. (04)]. The clinicopathological data are presented in Table 1.

**TABLE 1:** The clinicopathological data of 20 CRC patients.

Variables		Number
Age (years)	≥60	12
	<60	8
Gender	Male	12
	Female	8
Primary location	Right-sided tumor	9
	Left-sided tumor	9
	Rectal cancer	2
Histological Grade	Well-differentiated	2
	Moderately	18
T stage (TNM stage)	T1-2	3
	T3-4	17
Lymph node metastasis	No	11
	Yes	9
Distant metastasis	No	18
	Yes	2
Clinical stage	I-II	9
	III-IV	11
Microsatellite instability-high	Negative	17
	Positive	3

### 2.2 High-throughput sequencing

Additionally, a subset of four paired CRC tissue samples from Peking University Shenzhen Hospital was utilized for high-throughput sequencing conducted by Illumina PE150 (HaploX Genome Center, Shenzhen, China). The circRNA data were analysed using R language with the “limma” package. A heatmap was generated using the “pheatmap” package in R language, and a volcano plot was created via the Wei Sheng Xin website (http://www.bioinformatics.com.cn/plot_enhanced_volcano_plot_138) (Tang et al., 2023). Thresholds were set at |log_2_ fold change| >1 and p value < 0.05.

### 2.3 Fluorescence *in situ* hybridization (FISH)

The HT-29 and LoVo cells were used to perform FISH experiment. These cells were cultured in 20-mm confocal culture dishes. When the cells reached a density of 30%–50%, they were fixed at room temperature for 10 min using 4% paraformaldehyde solution (2 mL per well). Subsequently, 0.5% TritonX-100 PBS solution (1 mL per well) pre-cooled at 4°C was added to the cell culture dish and left standing for 5 min (at 4°C). Then, 200 μL of pre-hybridization solution was prepared for each well, and the cells were mixed with it, and blocked at 37°C for 30 min 200 μL of hybridization solution containing 5 μL of the probe was also prepared for each well and added to the cell culture dish, and incubated in the dark at 37°C overnight. Then, a 20×SSC hybridization washing solution was prepared (8.765 g of NaCl +4.41 g of sodium citrate, made up to 50 mL with ddH_2_O and adjusted to a pH of 7.0). Under dark conditions, the cells were washed three times with 4 × SSC hybridization washing solution at 42°C for 5 min each time, twice with 2 × SSC hybridization washing solution at 42°C for 5 min each time, and once with 1 × SSC hybridization washing solution at 42°C for 5 min, and once with 1 × PBS for 5 min. Finally, the cells were stained with 100 μL/well of buffer containing 1 × DAPI. After washing three times with 1 × PBS in the dark, the cells were fixed and observed and recorded under a microscope (Leica Microsystems, Wetzlar, Germany). The probe sequence of Cy3-labelled hsa_circ_0002238 was 5′ CY3-TCTTTCTCACTGATGTCCACTCTAA-3′ CY3 (GENESEED, Guangzhou, China). This probe was synthesized by GENESEED (https://www.geneseed.com.cn/) and specifically verified by the company.

### 2.4 siRNA and plasmid transfection

The siRNA sequence for hsa_circ_0002238 (GENESEED, Guangzhou, China) is presented in Table 2. Cells were cultured in 6-well plates prior to transfection. When they reached 70%–90% confluence, CRC cells were transfected with siRNA according to Lipofectamine 2000 protocol (Invitrogen, Shanghai, China). After 6 hours post-transfection, fresh medium was added to the cells, and Real-time quantitative polymerase chain reaction (qRT-PCR) was performed after 48 h to verify transfection efficiency.

**TABLE 2:** The siRNA sequence of hsa_circ_0002238.

siRNA	Sequence
si-hsa_circ_0002238–1	5′-GGA​CAU​CAG​UGA​GAA​AGA​UTT-3′
si-hsa_circ_0002238–2	5′-GAC​AUC​AGU​GAG​AAA​GAU​CTT-3′
si-hsa_circ_0002238–3	5′-CAU​CAG​UGA​GAA​AGA​UCA​GTT-3′

In addition, using an In-fusion kit (YEASEN, Shanghai, China), the hsa_circ_0002238 sequence was inserted into pLC5-ciR (GENESEED, Guangzhou, China), with TCC​TCT​CTT​GAT​TTC​CTT​ATT serving as the primer for pLC5-ciR. Subsequently, the prepared lentiviral solution (GENESEED, Guangzhou, China) was transduced into CRC cells. When 5∙10^5 CRC cells cultured in a 6-well plate reached 50%–60% confluence, 800 μL of fresh medium was added, followed by 200 μL of the lentiviral solution according to the multiplicity of infection to infect the cells. The medium was replaced with fresh medium after 12 h. Cell lysates were collected after 48 h, and qRT-PCR was performed to verify the infection efficiency.

### 2.5 Cell Counting Kit-8 (CCK-8) assay and flow cytometry analysis

For cell viability assessment via CCK-8 assay, approximately 5∙10^3 transfected cells were cultured in 96-well plates and treated with Cell Counting Kit-8 reagent (DOJINDO, Shanghai, China). 10 µL of CCK-8 solution were used to incubate the cells for 4 h. Absorbance readings at each time point-0 h, 24 h, 48 h, 72 h, and 96 h-were measured using a microplate reader at a wavelength of 450 nm (Thermo Fisher Scientific, Waltham, MA, United States).

For flow cytometry analysis, cells were maintained in medium supplemented with 10% FBS (Hyclone, Logan, Utah, United States), along with 100 U/mL penicillin (Hyclone, Logan, Utah, United States) and 100 U/mL streptomycin (Hyclone, Logan, Utah, United States). Following a transfection period of 48 h, cells from the 6-well plates were collected and stained with 5 μL each of Annexin V-FITC and Propidium Iodide solutions. According to protocols outlined by the Annexin V-FITC Apoptosis Detection Kit (Beyotime, Shanghai, China), detection occurred on an Analytical flow cytometer (Santa Clara, California, United States) 15 min later.

### 2.6 Wound healing and transwell assay

In wound healing assays, approximately 3.5∙10^5 transfected cells were seeded into 6-well plates. Once nearly confluent across culture plates, a straight line was drawn on them using a pipette tip. After PBS washing three times, the cell growth at 0 h, 24 h and 48 h was observed and recorded by photos using an inverted microscope (Olympus, Tokyo, Japan).

For transwell assay, 50 μL of matrigel matrix (BD Biocoat, Shanghai, China) diluted 5 times by FBS-free medium was added into the transwell chamber and incubated in a 37°C incubator for 30 min. About 3∙10^4 cells were added into the transwell chamber, and 600 µL of culture medium containing 10% FBS was added into the lower chamber of the transwells. After 24 h, the cells were fixed with 4% paraformaldehyde fixed solution (Servicebio, Wuhan, China) and stained with crystal violet (Beyotime, Shanghai, China), and then three photos were randomly taken under the microscope.

### 2.7 qRT-PCR

According to TsingZol Total RNA Extraction Reagent (TSINGKE, Beijing, China), total RNA was extracted from CRC cell lines and CRC tissues. The value of A260/A280 was detected by an ultra-micro spectrophotometer (KAIAO, Beijing, China), which was helpful to calculate the purity and concentration of RNA. cDNA was synthesized using SynScript™ III cDNA Synthesis Mix (TSINGKE, Beijing, China) and 2 × TSINGKE^®^ Matser qPCR Mix (SYBR Green I) (TSINGKE, Beijing, China) were used to perform PCR reaction. The primers of hsa_circ_0002238 and GAPDH are shown in Table 3. The 2^−ΔΔCt^ method was used to clarify the relative expression level of the DNA.

**TABLE 3:** The primers of hsa_circ_0002238 and GAPDH.

Gene		Sequence (5′-3′)
hsa_circ_0002238	Forward Primer	GCT​TAG​AGT​GGA​CAT​CAG​TG
	Reverse Primer	AAG​CCA​TCG​GTG​TTT​GTT​TC
GAPDH	Forward Primer	TCA​AGA​AGG​TGG​TGA​AGC​AGG
	Reverse Primer	TCA​AAG​GTG​GAG​GAG​TGG​GT

### 2.8 Western blot

The protein expression level was determined by Western blot. Firstly, RIPA lysis solution (Beyotime, Shanghai, China) and PMSF (Beyotime, Shanghai, China) were used to lyse CRC cells or tissues. The protein expression concentration was calculated based on BCA Protein Assay Kit (Beyotime, Shanghai, China). Secondly, 10% SDS-PAGE gels were used to perform electrophoresis with 80 V (60 min) and 120 V (30 min) in accordance with electrophoresis distance. Protein samples and protein markers (Fermentas, Waltham, Massachusetts, United States) were injected into the loading hole. Thirdly, the protein on the gels was transferred onto PVDFs (Millipore, Massachusetts, United States) and the PVDFs were sealed by TBST solution. Fourthly, the PVDFs with specific protein were incubated with primary antibody for at least 8h, and after being washed by TBST solution for 3 times, the PVDFs were incubated with secondary antibody for 1 h. Lastly, The PVDF was contacted with Enhanced Luminol Reagent (Thermo, Waltham, Massachusetts, United States) and Oxidizing Reagent (Thermo, Waltham, Massachusetts, United States) for 1–2 min and the figure could be observed by gel imaging system (Bio-rad, California, United States). Specific antibody dilution ratios are as follows: GAPDH (Proteintech, Chicago, United States, 1:5000), E-cadherin (Affinity, Cincinnati, Ohio, United States, 1:1000), N-cadherin (Affinity, Cincinnati, Ohio, United States, 1:1000), Vimentin (Affinity, Cincinnati, Ohio, United States, 1:1000), β-catenin (Proteintech, Chicago, United States, 1:5000), serine/threonine kinase (AKT) (Proteintech, Chicago, United States, 1:5000), p-AKT (Massachusetts, United States, 1:1000), caspase 3 (Proteintech, Chicago, United States, 1:1000), bcl-2 (Cambridge, England, 1:1000), Bax (Wuhan Sanying, China, 1:1000), PI3K (Wuhan Sanying, China, 1:1000), p-PI3K (Wuhan Sanying, China, 1:1000), anti-rabbit secondary antibody (Jackson, Pennsylvania, United States, 1:5000), anti-mouse secondary antibody (Jackson, Pennsylvania, United States, 1:5000).

### 2.9 *In vivo* studies

Six 5-week-old immunocompromised nude mice (Vital River, Beijing, China) were included in this study, and animal experiments were carried out in accordance with the guidelines of animal ethics institutions. First, a stable HT-29 cell line was constructed, including shRNA and sh-hsa_circ_0002238, and puromycin (Solarbio science & technology, Beijing, China) was added according to different concentration gradients. On the 7th day of cell culture, the drug concentration (5 μg/mL) when the cell lethality was 100% was screened as the drug concentration for subsequent experiments. Lentivirus and 5 μg/mL puromycin were successively added to cells with a density of 70% to continue screening cells (Stewart et al., 2003; Chumakov et al., 2010). The concentration of the constructed stable cell suspension was adjusted to 1∙10^6/mL, and 1∙10^5 cells were injected into the left axilla of nude mice. The tumor volume was measured by v ernier caliper every 3 days. Mice were euthanized 21 days after tumor implantation, and tumor weight was determined.

### 2.10 Statistical analysis

Figure generation was performed by GraphPad Prism 8.0.2 (GraphPad Software, Inc., San Diego, CA, United States) and student’s t-test were used to determine differences between groups. All data are presented as the mean ± SD. The ratio of the actual expression level of hsa_circ_0002238 in CRC cancer tissues to that in adjacent tissues was calculated. According to the calculated ratio, the median value was taken, and the clinical data of the patients were divided into two groups. The chi-square test was used to analyze the relationship between different groups. The Receiver Operating Characteristic curve (ROC curve) was used to evaluate the diagnostic value of the expression level of hsa_circ_0002238 for CRC. A p value less than 0.05 was considered to indicate a statistically significant difference (* indicates p value less than 0.05, ** indicates p value less than 0.01, *** indicates p value less than 0.001, **** indicates p value less than 0.0001).

## 3 Results

### 3.1 Hsa_circ_0002238 is highly expressed in CRC tissue and CRC cell lines

**FIGURE 1:**
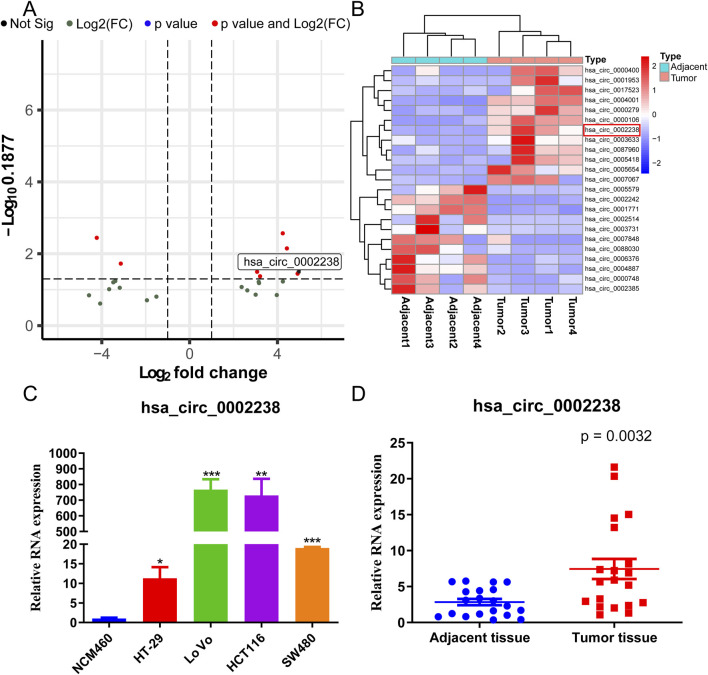
The expression level of hsa_circ_0002238 in CRC. **(A)** The volcano plot of differentially expressed circRNA in CRC high-throughput RNA sequencing dataset. **(B)** The heatmap of differentially expressed circRNA in CRC high-throughput RNA sequencing dataset. **(C)** The expression level of hsa_circ_0002238 in normal colon cells and CRC cells detected by qRT-PCR. **(D)** The expression level of hsa_circ_0002238 in CRC adjacent and tumor tissue detected by qRT-PCR. * indicates p value < 0.05, ** indicates p value < 0.01, *** indicates p value < 0.001. Abbreviation used: CRC, colorectal cancer; Not Sig, no significant statistical differences; Log2(FC), Log_2_ (Fold Change).

High-throughput sequencing was performed to identify circRNA with |log_2_ fold change| >1, consisting of 23 circRNA. Among them, hsa_circ_0002238 was highly expressed in CRC tissues (Figures 1A,B). Normal colon cells line NCM460 and colorectal cancer cell lines HT-29, LoVo, HCT116, SW480 were used to detect the expression level of hsa_circ_0002238 (Figure 1C). As illustrated, hsa_circ_0002238 was highly expressed in colorectal cancer cells, with HT-29 cells being the lowest and LoVo cells being the highest. Therefore, HT-29 cell line was utilized to upregulate hsa_circ_0002238, while LoVo cell line was used to downregulate hsa_circ_0002238 for the subsequent functional phenotype analysis. To further verify the expression level, 20 CRC adjacent tissues and 20 CRC tumor tissues were collected from Peking University Shenzhen Hospital. We validated that the expression level of hsa_circ_0002238 in tumor tissue was higher than that in adjacent tissue using qRT-PCR (Figure 1D).

**FIGURE 2:**
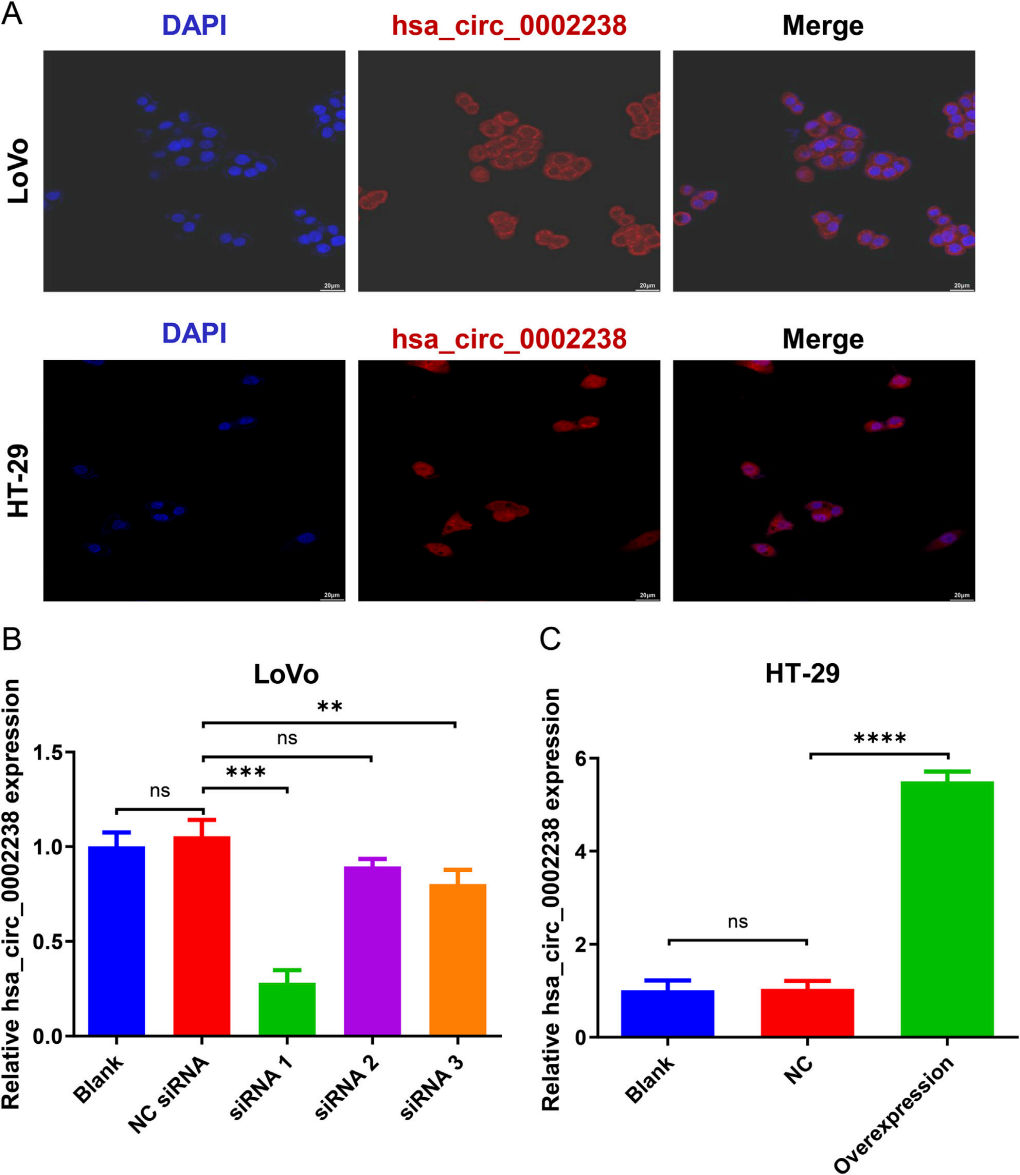
The verification of hsa_circ_0002238 expression level in CRC cells. **(A)** The location of hsa_circ_0002238 in LoVo and HT-29 cells determined with FISH experiment. **(B)** The expression level of hsa_circ_0002238 in LoVo cells after siRNA transfection detected by qRT-PCR. **(C)** The expression level of hsa_circ_0002238 in HT-29 cells after plasmid transfection detected by qRT-PCR. ns indicates no significant statistical differences, ** indicates p value < 0.01, *** indicates p value < 0.001, **** indicates p value < 0.0001. Abbreviation used: Blank, negative control group without any intervention; DAPI, 2-(4-amidinophenyl)-6-indolecarbamidine dihydrochloride; FISH, fluorescence *in situ* hybridization; NC, negative control group of hsa_circ_0002238 overexpression using plasmid; NC siRNA, negative control group of hsa_circ_0002238 knocked down using siRNA; ns, no significant statistical differences.

In addition, according to the FISH experiment, hsa_circ_0002238 was expressed in the LoVo and HT-29 cell cytoplasm (Figure 2A). After siRNA or plasmid transfection, the expression level of hsa_circ_0002238 was determined by qRT-PCR, showing that siRNA 1 (Figure 2B) and the plasmid (Figure 2C) had good transfection efficiency.

### 3.2 The relationship between the expression level of hsa_circ_0002238 and the clinicopathological characteristics of CRC patients and its diagnostic value

**FIGURE 3:**
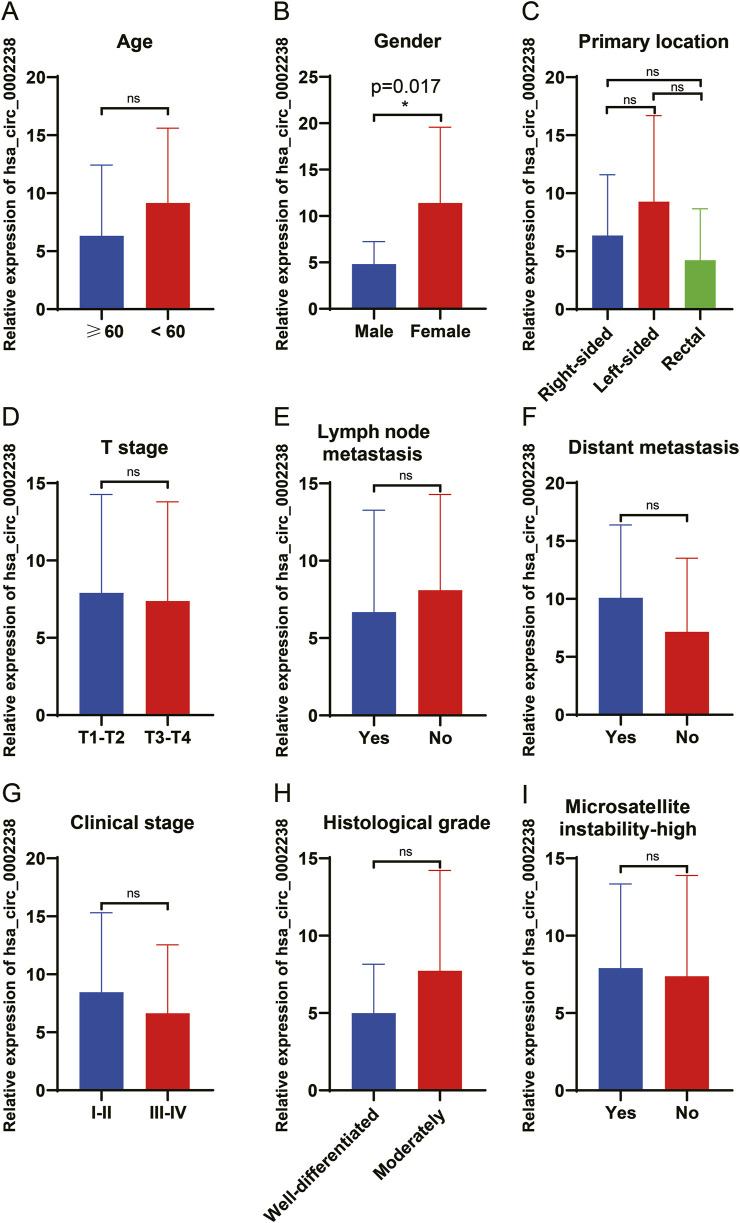
The relationship between the clinicopathological data of 20 CRC patients and the expression level of hsa_circ_0002238. The relationship between **(A)** age (years), **(B)** gender, **(C)** primary location, **(D)** T stage (TNM stage), **(E)** lymph node metastasis, **(F)** distant metastasis, **(G)** clinical stage, **(H)** histological grade and **(I)** microsatellite instability-high of 20 CRC patients and the expression level of hsa_circ_0002238. ns indicates no significant statistical differences, * indicates p value < 0.05.

**FIGURE 4:**
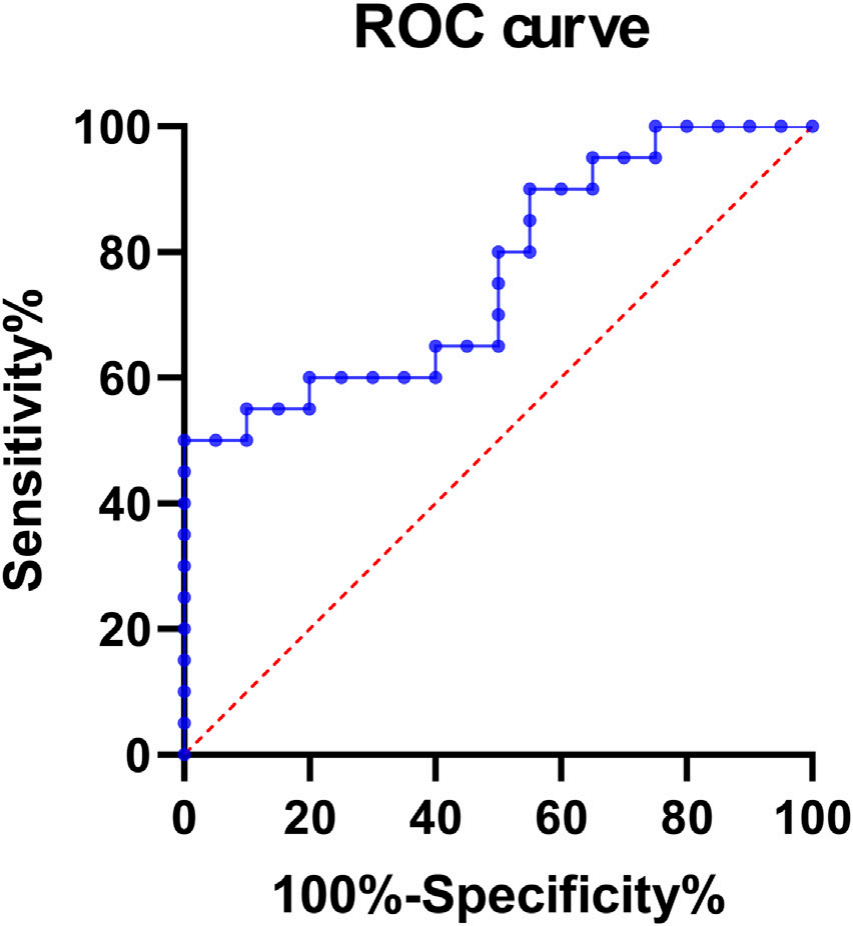
The ROC curve of the expression level of hsa_circ_0002238 in 20 CRC patients. The area under the ROC curve was 0.765 (95%CI: 0.618–0.913, p = 0.004). When the relative expression level of hsa_circ_0002238 was 5.836, the sensitivity (50%) and specificity (100%) for diagnosing CRC are both relatively high. Abbreviation used: ROC, Receiver Operating Characteristic.

In order to explore the relationship between hsa_circ_0002238 and the clinicopathological characteristics of patients as well as its diagnostic value, this study further collected the clinicopathological characteristic data of CRC patients and carried out data analysis. As can be seen from the results in Table 4, the gender of the patients was related to the expression level of hsa_circ_0002238 (p = 0.020), while there was no significant correlation between the expression level of hsa_circ_0002238 and other clinicopathological data of the patients. At the same time, box plots were drawn according to different clinical characteristics of the patients. The results showed that the expression level of hsa_circ_0002238 in the cancer tissues of female CRC patients was significantly upregulated compared with that in male patients, with a statistically significant difference (p = 0.017) (Figure 3B). However, other clinicopathological characteristics of CRC patients have no obvious relationship with the expression level of hsa_circ_0002238 (Figure 3). In addition, the value of the expression level of hsa_circ_0002238 for the diagnosis of CRC was preliminarily explored through the ROC curve (Figure 4). The results showed that the area under the ROC curve was 0.765 (95%CI: 0.618–0.913, p = 0.004), indicating that the expression level of hsa_circ_0002238 has good diagnostic accuracy. Among them, when the relative expression level of hsa_circ_0002238 was 5.836, the sensitivity (50%) and specificity (100%) for diagnosing CRC are both relatively high. Therefore, this study shows that the expression level of hsa_circ_0002238 is significantly correlated with the different genders of CRC patients, and the preliminary results indicate that hsa_circ_0002238 has a relatively high diagnostic value.

**TABLE 4:** The relationship between the clinicopathological data of 20 CRC patients and the expression level of hsa_circ_0002238.

Clinical characteristics		Expression level of hsa_circ_0002238		p value
		High (number)	Low (number)	
Age (years)	≥60	4	8	0.170
	<60	6	2	
Gender	Male	3	9	0.020
	Female	7	1	
Primary location	Right-sided tumor	4	5	0.895
	Left-sided tumor	5	4	
	Rectal cancer	1	1	
Histological grade	Well-differentiated	1	1	0.999
	Moderately	9	9	
T stage (TNM stage)	T1-2	3	0	0.211
	T3-4	7	10	
Lymph node metastasis	No	5	6	0.999
	Yes	5	4	
Distant metastasis	No	9	9	0.999
	Yes	1	1	
Clinical stage	I-II	5	4	0.999
	III-IV	5	6	
Microsatellite instability-high	No	8	9	0.999
	Yes	2	1	

### 3.3 Hsa_circ_0002238 promotes CRC cell proliferation and suppresses apoptosis

**FIGURE 5:**
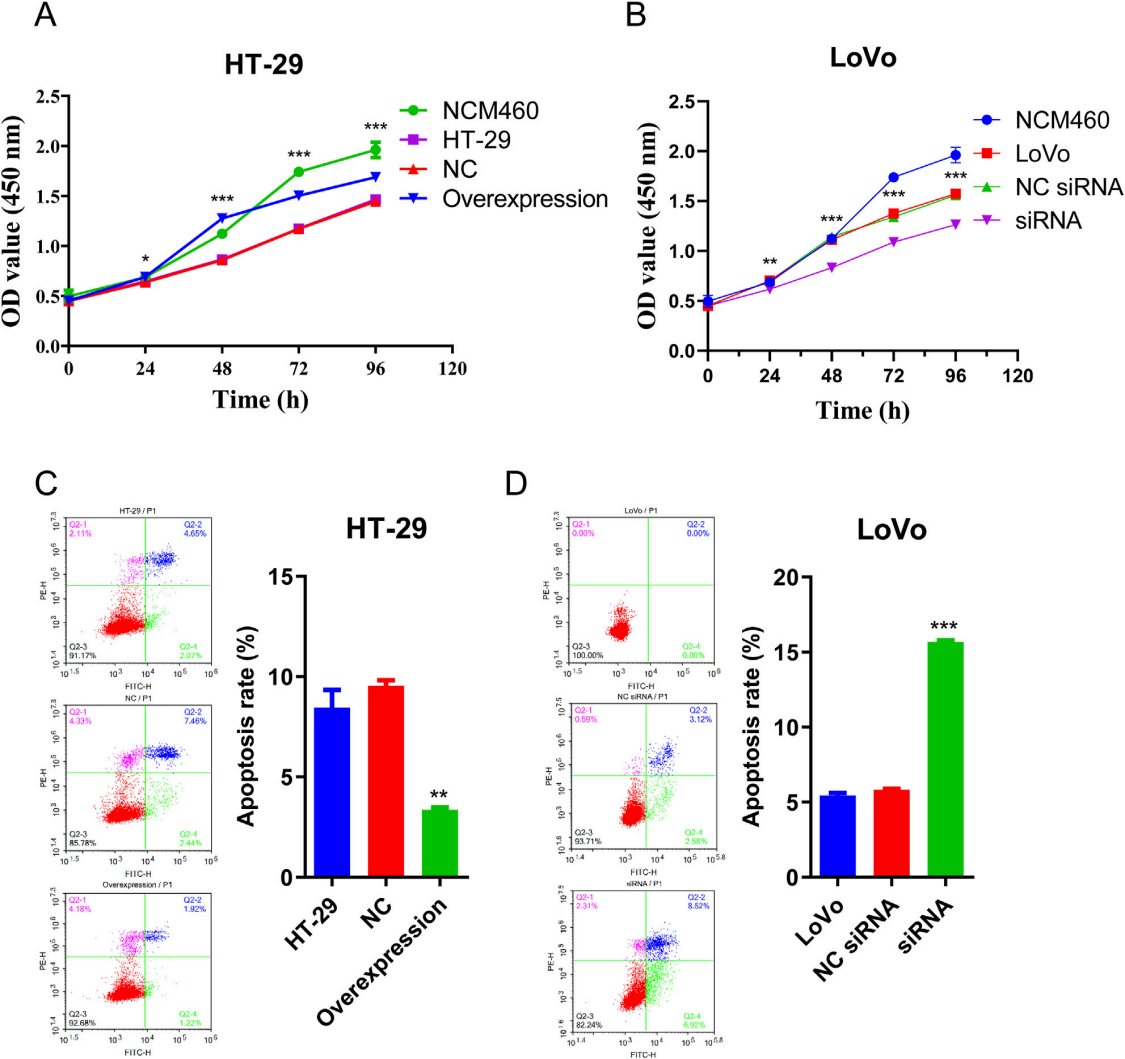
Hsa_circ_0002238 promotes CRC cell proliferation and suppresses apoptosis. **(A)** The proliferative capacity of HT-29 cells after hsa_circ_0002238 overexpression detected by CCK-8 assay. **(B)** The proliferative capacity of LoVo cells after hsa_circ_0002238 knockdown detected by CCK-8 assay. **(C)** The apoptosis rate of HT-29 cells after hsa_circ_0002238 overexpression detected by flow cytometry analysis. **(D)** The apoptosis rate of LoVo cells after hsa_circ_0002238 knockdown detected by flow cytometry analysis. * indicates p value < 0.05, ** indicates p value < 0.01, *** indicates p value < 0.001. Abbreviations used: NC, negative control group of hsa_circ_0002238 overexpression using plasmid; NC siRNA, negative control group of hsa_circ_0002238 knocked down using siRNA.

CCK-8 assay was conducted to evaluate CRC cell proliferation ability and flow cytometry analysis was used to detect apoptosis rate of CRC cells. As depicted in Figure 5A, the proliferation of HT-29 cells increased after hsa_circ_0002238 overexpression compared to the control group and colorectal normal cells. Conversely, knockdown of hsa_circ_0002238 decreased the proliferation of LoVo cells (Figure 5B). In Figures 5C,D, overexpression of hsa_circ_0002238 reduced the apoptosis rate of HT-29 cells, while downregulation of hsa_circ_0002238 enhanced that of LoVo cells. Therefore, hsa_circ_0002238 can promote CRC cell proliferation and inhibit apoptosis.

### 3.4 Hsa_circ_0002238 promotes CRC cell migration and invasion

**FIGURE 6:**
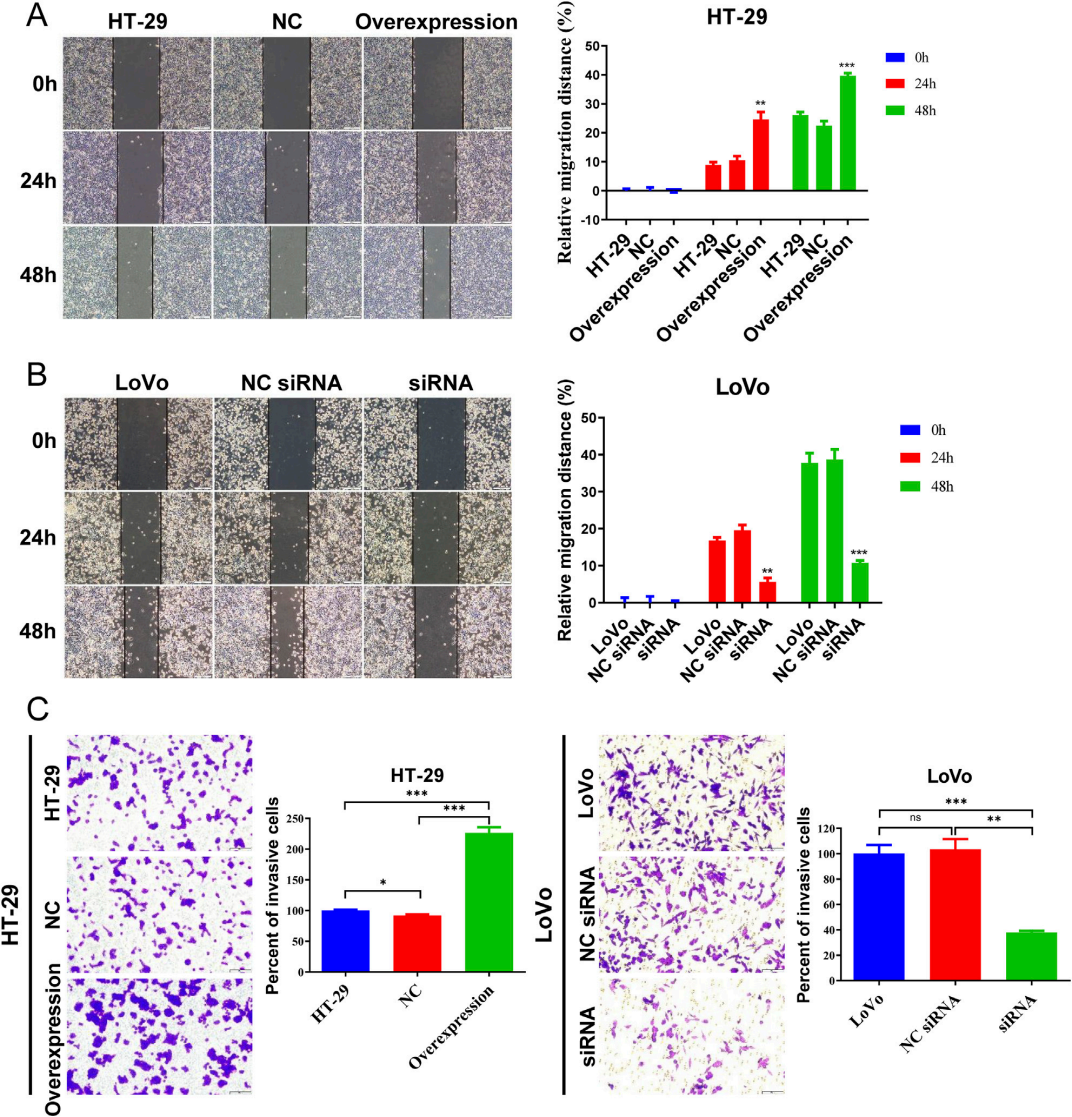
Hsa_circ_0002238 promotes CRC cell migration and invasion. **(A)** The migratory capacity of HT-29 cells after hsa_circ_0002238 overexpression determined with wound healing assays. **(B)** The migratory capacity of LoVo cells after hsa_circ_0002238 knockdown determined with wound healing assays. (**C**) The invasive ability of HT-29 cells after hsa_circ_0002238 overexpression and LoVo cells after hsa_circ_0002238 knockdown determined with transwell assays. ns indicates no significant statistical differences,* indicates p value < 0.05, ** indicates p value < 0.01, *** indicates p value < 0.001. Abbreviations used: NC, negative control group of hsa_circ_0002238 overexpression using plasmid; NC siRNA, negative control group of hsa_circ_0002238 knocked down via siRNA.

Wound healing and transwell assay were utilized to assess CRC cell migration and invasion ability. In the wound healing assay, more HT-29 cells with hsa_circ_0002238 overexpression migrated at 24 h and 48 h (Figure 6A), whereas fewer LoVo cells with hsa_circ_0002238 downregulation migrated at 24 h and 48 h (Figure 6B). Similarly, compared with the control group, the invasion ability of the HT-29 cell line increased after hsa_circ_0002238 overexpression through plasmid transfection, while the invasion capability of the LoVo cell line decreased after hsa_circ_0002238 knockdown (Figure 6C). Thus, hsa_circ_0002238 can promote CRC cell migration and invasion.

### 3.5 Knockdown of hsa_circ_0002238 inhibits CRC tumor growth *in vivo*

**FIGURE 7:**
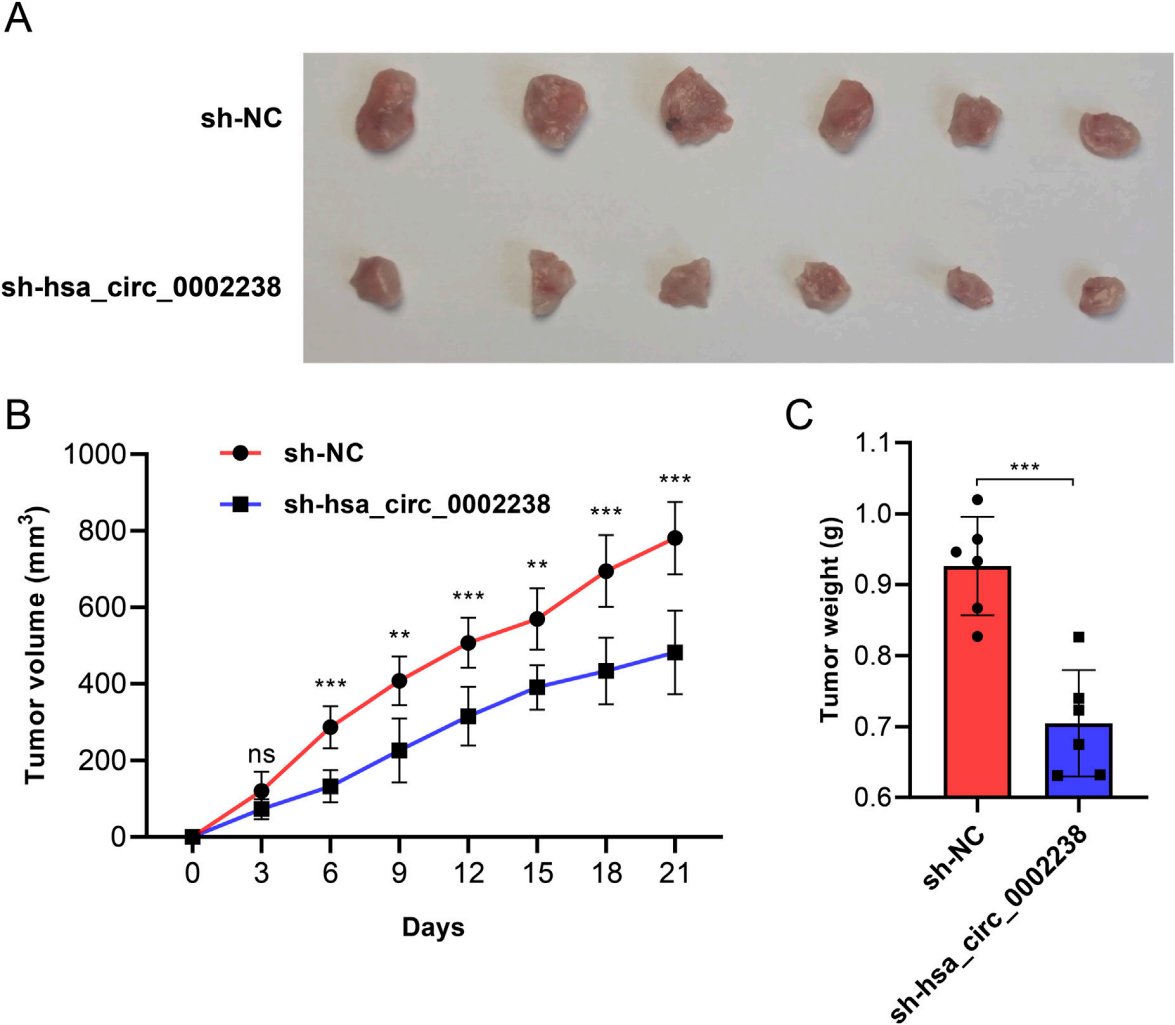
Knockdown of hsa_circ_0002238 inhibits CRC tumor growth *in vivo*. **(A)** Representative photographs of CRC tumor tissues of mice in each group. **(B)** The volume of mice in each group at different time points after inoculation with CRC tumors. **(C)** The weight of CRC tumors of mice in each group. ns indicates no significant statistical differences, ** indicates p value < 0.01, *** indicates p value < 0.001. Abbreviations used: CRC, colorectal cancer; sh-hsa_circ_0002238, group of hsa_circ_0002238 knocked down via shRNA. sh-NC, negative control group of hsa_circ_0002238 knocked down via shRNA.

To investigate the effect of hsa_circ_0002238 on CRC tumor growth *in vivo*, HT-29 cell lines stably transfected with sh-NC or sh-hsa_circ_0002238 were constructed. Figure 7A indicated that compared with the sh-NC group, the CRC tumor growth in the sh-hsa_circ_0002238 group was significantly reduced. The tumor volume was measured at each time point and conducted statistical analysis. The results showed that the tumor volume of the sh-hsa_circ_0002238 group increased more slowly than that of the control group on days 6, 9, 12, 15, 18, and 21 (Figure 7B). In addition, the tumor weight of CRC in mice on day 21 was assessed, revealing a significant reduction in the sh-hsa_circ_0002238 group compared to the sh-NC group (Figure 7C). Consequently, knockdown of hsa_circ_0002238 resulted in a suppression of CRC tumor growth in mice.

### 3.6 Hsa_circ_0002238 promotes epithelial-mesenchymal transition (EMT), PI3K/AKT pathway and suppresses apoptosis in CRC cells

**FIGURE 8:**
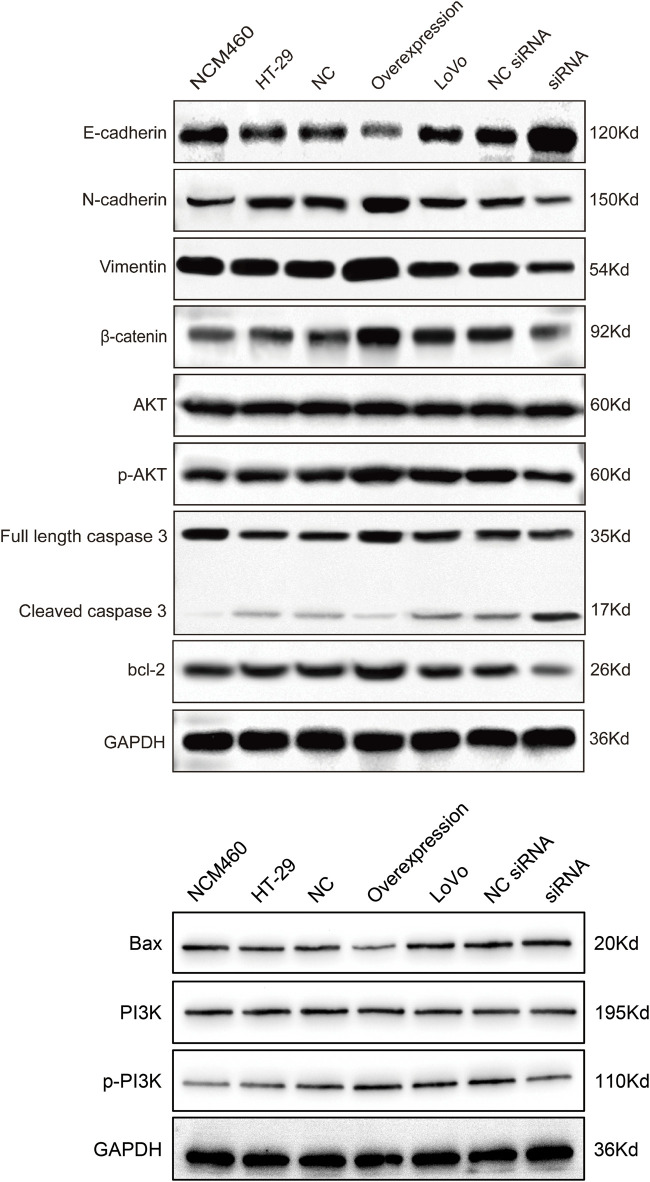
Western blot analysis of the expression level of epithelial‐mesenchymal transformation-related markers, PI3K/AKT signaling pathway-related markers and apoptosis-related markers after hsa_circ_0002238 abnormal expression in CRC cells.

**FIGURE 9:**
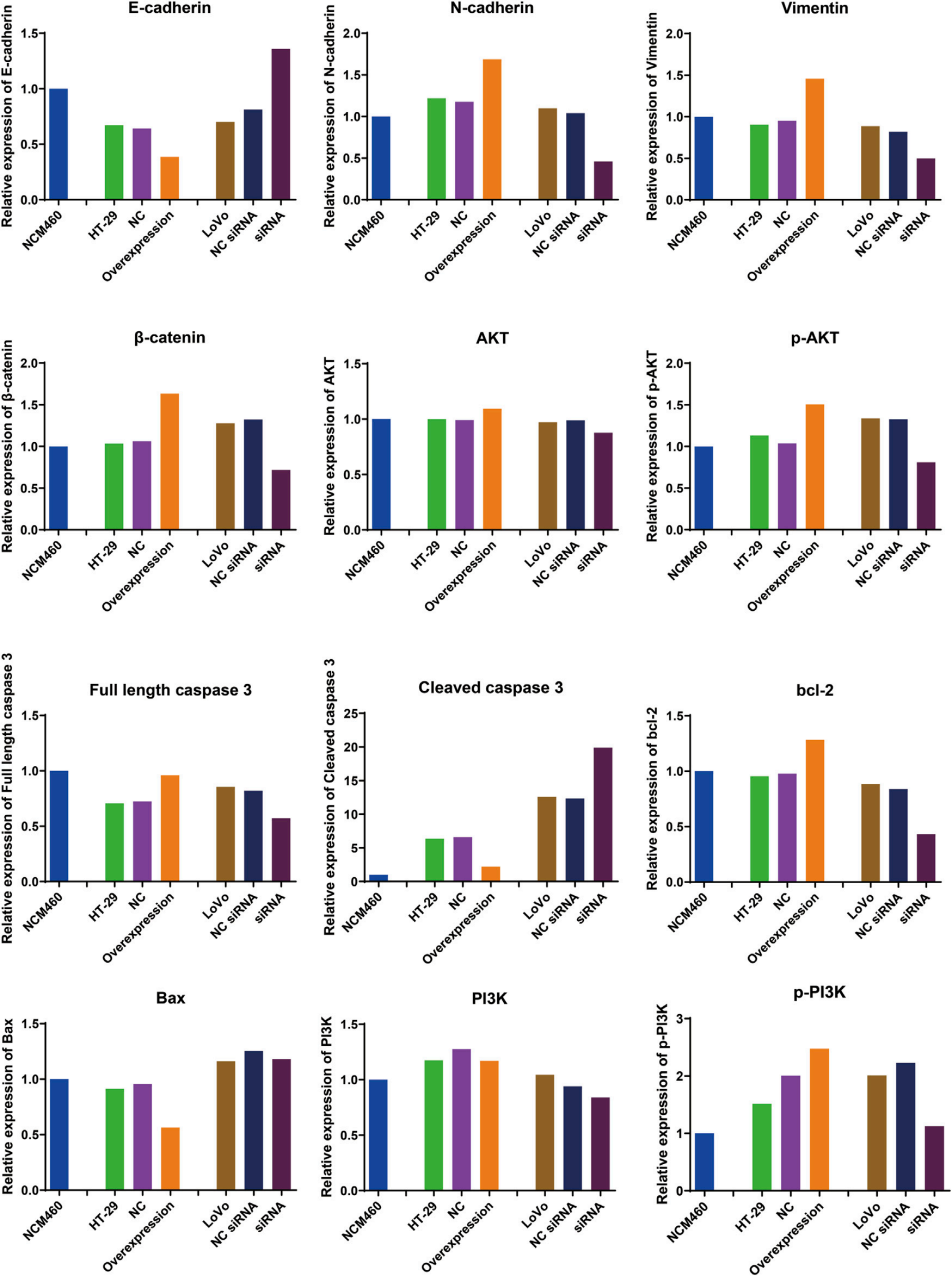
Quantitative analysis results of Western blot for the expression level of epithelial-mesenchymal transition-related markers, PI3K/AKT signaling pathway-related markers and apoptosis-related markers after hsa_circ_0002238 abnormal expression in CRC cells.

The EMT process can stimulate the transformation of epithelial cells into fibroblasts or mesenchymal cells with deficient cell polarity and cytoskeletal rearrangement, which regulates cancer tumorigenesis, metastasis, immunosurveillance, drug resistance, etc (Cao, 2024). E-cadherin is epithelial cell marker (Hui San and Ching Ngai, 2024), while N-cadherin and vimentin are common mesenchymal cell markers (Wang X. et al., 2024). β-catenin possesses the ability to form a complex with E-cadherin, thereby enhancing the intercellular connections and fostering cellular cohesion (Hülsken et al., 1994). To investigate whether hsa_circ_0002238 regulates CRC development through EMT process, specific protein were analyzed via Western blot. In Figures 8, 9, the expression level of E-cadherin in HT-29 cells with hsa_circ_0002238 overexpression was low, while it was high in LoVo cells with hsa_circ_0002238 knockdown. Conversely, N-cadherin, vimentin and β-catenin were upregulated in HT-29 cells with hsa_circ_0002238 overexpression and downregulated in LoVo cells with hsa_circ_0002238 knockdown compared to the control groups, respectively. These findings suggest that hsa_circ_0002238 promotes EMT process in CRC cells. Furthermore, p-AKT and p-PI3K was highly expressed in HT-29 cells with hsa_circ_0002238 overexpression, whereas it decreased in LoVo cells with hsa_circ_0002238 knockdown, suggesting that the expression level of hsa_circ_0002238 may be related to the PI3K/AKT signaling pathway and enhance CRC growth. The caspase family is a group of highly efficient and specific proteases that mediate the hydrolysis of dying cells, and the bcl-2 family mainly plays an inhibitory role in apoptosis (Zhang X. et al., 2024). In Figures 8, 9, it is clear that cleaved caspase 3 and bcl-2 was decreased, while Bax was highly expressed in HT-29 cells with hsa_circ_0002238 overexpression, indicating that hsa_circ_0002238 enhanced the apoptosis of CRC cells.

## 4 Discussion

CRC is one of the cancers with the highest morbidity and mortality rates globally (Bray et al., 2024). Early detection and accurate diagnosis represent pivotal strategies in the management of CRC, significantly enhancing the survival rates of affected patients. The advent of carcinoembryonic antigen (CEA) testing, alongside the widespread adoption of colorectal endoscopy in clinical practice, has indeed improved early diagnostic capabilities for CRC to some extent. However, numerous limitations persist. For instance, while CEA exhibits a specificity rate of 91.1% in CRC cases, its sensitivity remains disappointingly low at merely 54.7% (Sun and Long, 2024). Furthermore, many individuals do not undergo regular colorectal endoscopies due to various factors, which makes it difficult to identify CRC at an early stage (Mannucci and Goel, 2024). Consequently, there is an urgent need to discover more molecular markers that can facilitate earlier diagnoses of CRC. Advances in molecular biology technologies have opened new avenues for identifying additional biomarkers. For instance, membrane palmitoylated protein has emerged as an independent prognostic risk factor for CRC. Intriguingly, its expression is inversely regulated by DNA methylation processes (Yang et al., 2024). In addition, miRNAs, known for their ease of detection and regulatory roles throughout all stages of tumor progression, hold significant promise for predicting patient prognosis during therapeutic interventions (Rac, 2024). In recent years, research into circRNAs as potential molecular markers for early cancer diagnosis has gained its popularity. For example, circ_0006949 identified in sputum demonstrates higher expression levels in non-small cell lung cancer and correlates with poor patient prognosis (Bai et al., 2024). Similarly, hsa_circRNA_000166 shows a positive correlation with tumor size, TNM staging, histological grading, and lymph node metastasis among breast cancer patients while exhibiting negative correlations with both progression-free survival and overall survival after surgery (Wang M. H. et al., 2024). Thus, exsiting implications suggest that circRNA possesses substantial potential as a biomarker within the realms of malignant tumor diagnosis and treatment strategies. Recently, the research of circRNA in CRC has also gradually increased (Zhou et al., 2024). Therefore, we conducted functional phenotype experiments of circRNA in CRC.

In this study, we identified a novel circRNA, hsa_circ_0002238, which exhibits significantly higher expression levels in CRC compared to adjacent non-cancerous tissue. Furthermore, hsa_circ_0002238 enhances proliferation, migration, and invasion in CRC cells. Although hsa_circ_0002238 has rarely reported in cancer, it has been found that other circRNAs affect the differentiation, invasion and metastasis of CRC. Previous research indicated that circ_0038718 was overexpressed in CRC tissue, facilitating CRC proliferation and migration (Li et al., 2024). Interestingly, silencing hsa_circ_0004194 led to the decrease of CRC tumor growth, tumor volume and liver metastasis, which inhibited CRC progression (Lin et al., 2024). Therefore, our research shows that hsa_circ_0002238 plays an important role in the proliferation, migration and invasion of CRC cells. In our study, flow cytometry analysis also revealed that the apoptosis rate was reduced after hsa_circ_0002238 overexpression. Additionally, with high expression of hsa_circ_0002238, there was a notable decrease in caspase 3, bcl-2 and an increase in Bax expression determined with Western blot, suggesting that CRC is inhibited from undergoing apoptosis. Similar circRNA findings can be discovered in the article published by Li et al., suggesting that circRNA CDR1as decreased apoptosis rate of CRC via regulating Bax, Bcl-2, caspase 3 and caspase 9 proteins (Li et al., 2023). In addition, another study also found that circRNA can cause CRC apoptosis (Yin et al., 2024). Therefore, our study confirmed that hsa_circ_0002238 is also one of the key molecules to regulate CRC apoptosis. Furthermore, our results suggest that an EMT process occurs within CRC cells exhibiting high levels of hsa_circ_0002238 expression. In cells exhibiting high expression levels of hsa_circ_0002238, a decrease in E-cadherin expression was observed, while the expressions of N-cadherin, vimentin and β-catenin were found to be elevated. Sun et al. also found that circ_0114866 knockdown upregulated MYL6B expression by sponging miR-653-5p, and inhibited the progression of non-small cell lung cancer and EMT process (Sun et al., 2024). Another study reported that circ_0006168 modified by N6-methyladenosine promoted EMT in esophageal squamous cell carcinoma through miR-384/STAT3/Snail axis (Wu et al., 2024). Therefore, hsa_circ_0002238 may promote EMT process in CRC. Concurrently, the expression of p-AKT and p-PI3K also increased alongside the heightened levels of hsa_circ_0002238. Recent study have proposed that circKDM1A activates the AKT signaling pathway by up-regulating PDK1 to promote the progression of CRC (Wu et al., 2024). It was reported that circRNA MALAT1 promoted the progression of intrahepatic cholangiocarcinoma by competing with miR-512-5p to bind to VCAM1, leading to upregulation of VCAM1 transcription and activation of PI3K/AKT signaling pathway (Zhang M. et al., 2024). Thus, these results indicates that alterations in hsa_circ_0002238 expression may also influence the PI3K/AKT signaling pathway. In conclusion, our findings show that hsa_circ_0002238 promotes proliferation, migration, invasion and inhibits apoptosis of CRC cells, as well as activates EMT process and PI3K/AKT signaling pathway. This study shows that the expression level of hsa_circ_0002238 is significantly associated with patient gender and its potential as a diagnostic biomarker for CRC, requiring further investigation to confirm its clinical utility and explore its molecular mechanisms.

However, this study has several limitations. Firstly, we only investigated the functional phenotypes of hsa_circ_0002238 in CRC, and its specific molecular mechanism remains to be elucidated. Secondly, we used a limited number of CRC cases, which may exist research bias. Thirdly, we did not evaluate the relationship between hsa_circ_0002238 expression level and prognosis of CRC patients. Therefore, future research will focus on further elucidating the specific molecular mechanisms by which hsa_circ_0002238 influences CRC proliferation, migration, invasion, apoptosis, EMT process and PI3K/AKT signaling pathway, which includes exploring aspects such as protein modification and miRNA sponging. Additionally, we will expand our sample size for CRC cases to validate the potential role of hsa_circ_0002238 in diagnosis and prognosis of CRC more comprehensively.

## 5 Conclusion

In summary, hsa_circ_0002238 is highly expressed in CRC cells and CRC tissue. We also demonstrate that hsa_circ_0002238 promotes proliferation, migration, invasion and inhibits apoptosis, as well as facilitates EMT process and PI3K/AKT pathways in CRC cells, suggesting its potential as a diagnostic biomarker for CRC simultaneously. Therefore, hsa_circ_0002238 plays a crucial role in the occurrence and development of CRC. This provides a theoretical foundation for future research on the specific molecular mechanism of hsa_circ_0002238 in CRC and the significance of hsa_circ_0002238 in diagnosis and prognosis of CRC.

## Data Availability

The original contributions presented in the study are publicly available. This data can be found here: https://figshare.com/articles/dataset/Original_data_zip/29246426/1.
